# When Does the Danger of Infection in Scarlatina Cease?

**Published:** 1882-03

**Authors:** 


### WHEN DOES THE DANGER OF INFECTION IN SCARLATINA CEASE?

Mr. John Simon (*Lancet*, vol. I, 1881, page 146) says: "It is believed that the dispersion of contagious dust from the patient's skin is impeded by keeping his entire body (including limbs and head and face) constantly anointed with oil, or other grease; and some practitioners also believe this treatment to be of advantage to the patient himself. When the patient's convalescence is complete, the final disinfection of his surface should be effected by warm baths, with abundant soap, taken three or four successive days, till no trace of roughness of the skin remains. Not until this has been done, nor without the greatest care that the clothes are clean, and free from infection, should the patient, however slight may have been the attack, be allowed to associate with persons susceptible to scarlatina."—*N. Y. Med. Times.*

